# Evaluation of reciprocal F1 crosses of Fayoumi with two exotic chicken breeds 2: additive and non-additive effects on egg quality traits

**DOI:** 10.1007/s11250-023-03728-8

**Published:** 2023-09-19

**Authors:** Fikrineh Negash, Solomon Abegaz, Yosef Tadesse, Temesgen Jembere, Wondmeneh Esatu, Tadelle Dessie

**Affiliations:** 1 Adami Tulu Agricultural Research Center, P. O. Box 35, Batu, Ethiopia; 2 School of Animal and Range Sciences, Haramaya University, P. O. Box 138, Dire Dawa, Ethiopia; 3 Ethiopian Institute of Agricultural Research, P. O. Box 2003, Addis Ababa, Ethiopia; 4 International Livestock Research Institute, P.O. Box 5689, Addis Ababa, Ethiopia

**Keywords:** Crossbreeding effect, Combining ability, Egg quality parameter, Fayoumi chicken breed, Reciprocal crossing

## Abstract

The current study evaluates additive and non-additive genetic variances for egg quality traits in six genotypes generated through pure mating and reciprocal crossing of Fayoumi (FM) with Koekoek (KK) and White Leghorn (WL). For each genotype, measurements were taken on 30 eggs randomly sampled at 32, 36, and 40 weeks of age to evaluate both external and internal egg quality parameters. The results revealed significant differences (*P* < 0.001) among the genotypes in all external quality traits and most internal quality traits, including yolk weight (YW), albumen weight (AW), and yolk height (YH). The results also showed that variations due to purebred effect (PE), general combining ability (GCA), maternal effect (ME), and specific combining ability (SCA) were significant in most traits, which reflects that both additive and non-additive variances are important for the inheritances of the parameters investigated. In most of the traits, the ME and PE were higher in KK and WL, while GCA was higher in KK and FM. The FM x WL had higher SCA than FM x KK. The results suggest the likelihood of genetic improvement in these genotypes through selection and crossbreeding strategies and/or a combination of the two.

## Introduction

In Ethiopia, the poultry improvement program, which aimed to enhance egg and meat production from indigenous chickens, was started in the early 1950s (Tadelle et al. 2000). The program mainly involved the importation of genetically superior exotic breeds for use as either purebred or to be crossed with unselected indigenous ones. As part of this effort, imported chickens were distributed to rural smallholder farmers through on-farm research and public extension systems. Until the agricultural research system started evaluating the performances of those breeds (FAO 2019), documented studies related to the adaptability of the genotypes to the local environment did not exist (Wondmeneh et al. 2014). The previous poultry development strategy, which aimed to increase the productivity of indigenous chickens, has historically had limited success, despite the paucity of empirical evidence to support it. Its failure is due to various factors, including poor adaptation of the breeds to a harsh production environment, their high susceptibility to disease challenges, and poor on-farm management practices (Tadelle et al. 2000; FAO 2019). The manifestation of this failure is that the contribution of exotic chickens to the total poultry population and egg production (for instance, 9.11 and 9.38% in 2020, respectively; CSA 2021) remains at less than 10% after more than half a century of the program’s inception.

In a situation where the environment is sub-optimal to allow expression of the potential of exotic breeds, systematically crossing them with locally adapted chicken breeds may ensure the concert of the genotype and the environment. The strategy could improve overall production efficiency by exploiting the breeds’ complementarity for high genetic merit in different traits (Simm et al. 2021). Crossbreeding also exploits additive and non-additive genetic variations. The additive variation explains the average effect of the strains/breeds or parental lines involved, weighted according to the level of each parental population in the crossbreds, while the non-additive variation describes gene combination (heterosis) effects (Galukande et al. 2013). Heterosis has long been utilized in poultry breeding programs to produce progenies that exhibit higher performances than the average of their parental breeds (Williams et al. 2002).

Crossbreeding brings genetic progress to the population, but the change is not always permanent (Galukande et al. 2013). Furthermore, regularly producing crossbred animals would not be sustainable due to the high cost of obtaining and maintaining exotic genetic materials required in such crossbreeding system (Leroy et al. 2016). Synthetic breed formation is an alternative to a regular crossing system to bring long-lasting genetic progress in the population (Galukande et al. 2013). It provides the advantage of maintaining only one locally adapted population with all desirable traits of the breeds involved instead of two or more purebred flocks required for regular crossbreeding (Munisi et al. 2015). Designing a synthetic breed formation program necessitates an evaluation of genetic variations (additive and non-additive) and a better understanding of the mode of genetic inheritance for different traits to identify genetic stocks that would have a good combining ability (Falconer and Mackay 1996).

Evaluating the breeds for their combining ability and estimation of crossbreeding effects requires systematic crossbreeding designs (Jakubec et al. 1987). Diallel crossing is one of these methods used in testing populations. Although the complete diallel is most efficient in giving detailed information about crossbreeding effects with a small number of breeds, it is demanding and costly when the number of parents increases (Wolf and Knížetová 1994). As a result, Wolf et al. (1991) recommended simpler experimental designs with fewer purebred and crossbred populations. They further noted that there are good reasons to split breeds used as parents into two sets, where only crosses between these sets are of interest.

Substantial variations have existed among the exotic chicken breeds that have been imported into the country since the 1950s and evaluated under intensive and village production systems. Recent studies that evaluated one or more of those breeds (e.g., Lemlem and Tesfay 2010; Geleta et al. 2013; Tadesse et al. 2013; Geleta and Abdulkadir 2018; Senbeta and Balcha 2020) demonstrated variations in performance among Fayoumi, Koekoek, and White Leghorn. For instance, the breeds laid 144 − 159, 187 − 213, and 173 eggs per year, respectively. Besides, they are recognized to have different genetic merits. Fayoumi is an indigenous dual-purpose breed of Egypt. This breed is known for its hardiness, resistance to some infectious (including Newcastle disease virus, Marek’s disease virus, Salmonella, and Eimeria) diseases, and adaptation to harsh production environments (Besbes 2009; Bekele et al. 2010), like local chickens of Ethiopia. However, the empirical evidence indicates its lower production performance than Koekoek (a large dual-purpose breed) and White Leghorn (an egg-type breed).

Reciprocal crossing of Fayoumi with Koekoek and White Leghorn would be worthwhile in passing on high-production genes from the latter two breeds to Fayoumi, which already has genes responsible for survival under harsh rural conditions. The reciprocal and purebred populations can be evaluated when the differences in gene frequencies are supposed to exist between sire and dam breeds. For these crosses, crossbreeding parameters such as mean, heterosis, general and specific combining ability, purebred and maternal effects, and residual reciprocal effects can be estimated as in a complete diallel (Jakubec et al. 1987; Wolf et al. 1991). This article is the follow-up of the first report that investigates the additive and non-additive genetic effects on egg production traits. The current study aimed to investigate additive and non-additive genetic effects on egg quality traits for genotypes generated through pure mating and reciprocal crossing of Fayoumi with Koekoek and White Leghorn.

## Materials and methods

### Study location

The experiment was carried out at the poultry research farm of Haramaya University, which is located at an elevation of 2010 m above sea level, 9°26' N latitude and 42°03' E longitude (Senbeta 2017). The rainfall pattern of the area is bimodal, with mean annual precipitation ranging from 600 to 1260 mm while mean temperatures ranging between 9.74 °C and 24.05 °C (Burga et al. 2020; Adem 2021).

### Mating plan and incubation

A total of 45 hens and 15 cocks of Fayoumi (FM) and 30 hens and 10 cocks of each Koekoek (KK) and White Leghorn (WL) were used as a base population for mating. Crossbreds were produced by artificially inseminating hens of KK and WL with semen collected from FM cocks, and inseminating the hens of the later breed with semen collected from cocks of KK and WL. Semen was collected in the afternoon using an abdominal massaging technique. Fresh pooled semen was then diluted using saline solution (0.9%) in a ratio of 1:3. The insemination was carried out twice a week by depositing 0.1 mL of diluted semen into the left oviduct of the hens using a pipette (Adeleke et al. 2012). Purebreds were produced by naturally mating hens of each breed with cocks of their type.

Eggs were collected daily and labeled based on the sire and dam of the mating to avoid a mix-up of eggs during hatching and chicks after hatching. Incubation was performed under a standard procedure using an incubator with 37.5 °C temperature and 55% relative humidity. Candling was performed on day 18 to identify fertile eggs. Eggs that contained living embryos were transferred to hatching trays and placed in a hatcher. At hatch, chicks were tagged and placed in brooding pens.

### Management of birds

The birds were raised in the brooding pens using infrared lamps as a heat source until 8 weeks, while they reared in the grower pens under natural lighting from 9 to 20 weeks as described previously (Negash et al., 2023). At 21 weeks, hens of each genotype were randomly distributed into three pens as replications under a completely randomized design (CRD). The birds were offered a measured quantity of feed and clean water ad libitum throughout the study period. An adjustment to the amount of feed offered was made every week based on the development stages of the birds (NRC 1994). From hatching to 8 weeks, a ration provided to chicks had 20% crude protein (CP) and 2800 kcal/kg metabolizable energy (ME), and then the birds' feed was changed into a grower ration having 16% CP and 2800 kcal/kg ME until 20 weeks of age. From 20 to 40 weeks, the birds were offered a layer ration (with 16% CP and 2750 kcal/kg ME). Vaccines against Newcastle (NCD; HB1 and Lasota), infectious bursal (Gumboro), and fowl pox diseases were administered to the birds at appropriate stages. Treatments were also administered to the birds using anti-coccidial and anti-helminthic medications.

### Measurements taken

Thirty fresh eggs (10 from each pen) were randomly sampled for each genotype at 32, 36, and 40 weeks of age to determine egg quality parameters (Rajaravindra et al. 2015). Egg weight (EW), egg length (EL), and egg width (EWD) were measured on unbroken eggs using appropriate tools (weighing balance for EW and digital caliper for EL and EWD). The EL and EWD were employed to determine the egg shape index (ESI) according to the following equation:

$$ESI\left(\%\right)=\frac{EWD}{EL}*100$$

The eggs were broken out on a flat glass to determine internal quality parameters. Albumen height (AH) and yolk height (YH) were measured using a tripod micrometer, and consequently, albumen and yolk were carefully detached to take Albumen weight (AW) and yolk weight (YW). Haugh unit (HU) was estimated based on AH and EW using the following equation:

$HU=log(AH-{1.7EW}^{0.37}+7.57)$ where, AH and EW are albumen height in mm and egg weight in grams, respectively (Haugh 1937).

Shell thickness (ST) was measured at the middle and each end of the eggshell (the broad and narrow ends) using a digital caliper, and an average of the three measurements was taken as ST values. The eggshell was washed gently using tap water, air-dried for 48 h, and weighed to determine shell weight (SW).

### Statistical analysis

Data were analyzed as repeated measures using the PROC MIXED model procedure of JMP (SAS Institute Inc. 2018) to determine the difference between the genotype (i.e., genetic group) with genotype and age (weeks) as the main factors. The mean differences among the genotypes were separated using Tukey’s HSD test. The Box-Cox transformation method (Box and Cox 1964) was applied to transform data for non-normally distributed traits. The statistical model used was:

$${Y}_{\mathrm{ijk}}=\mu +{G}_{\mathrm{i}}+{A}_{\mathrm{j}}+{(GA)}_{\mathrm{ij}}+ {e}_{\mathrm{ijk}}$$

where, ${Y}_{\mathrm{ijk}}$ is the measurement of egg quality traits on *k*th observation (pen) of the *i*th genetic group; $\mu$ is the overall population mean; ${G}_{\mathrm{i}}$ is the effect of the *i*th genetic group (*i* = 1—7); ${A}_{\mathrm{j}}$ is the effect of *j*th week; ${(GA)}_{\mathrm{ij}}$ is interaction effect of *i*th genetic group and *j*th week; and ${e}_{\mathrm{ijk}}$ is the random error term.

For the traits that showed significant differences among the genotypes, a diallel model developed by Henderson (1948) and applied by Harvey (1960)—a model recommended to be suitable for the analysis of both full and partial diallel experiments (Model B; Jakubec et al. 1987)—was employed to estimate genetic effects. Before this analysis, percentages were transformed to arcsine square root values. The effect of each parental breed was assumed as fixed, and hence all effects in the model were considered fixed effects (Nath et al. 2007). The statistical model used was:

$${y}_{\mathrm{hijk}}=\mu +{a}_{\mathrm{h}}+{p}_{\mathrm{ii}}+{g}_{\mathrm{i}}+{g}_{\mathrm{j}}+ {m}_{\mathrm{j}}+{s}_{\mathrm{ij}}+{r}_{\mathrm{ij}}+{e}_{\mathrm{hijk}}$$

where, ${y}_{\mathrm{hijk}}$ is the *k*th observation on the progeny of a mating between *i*th sire group and *j*th dam group in *h*th type of breeding (purebred or crossbred); $\mu$ is the overall population mean; ${a}_{\mathrm{h}}$ is an effect common to all progenies of *h*th type of breeding (purebred or crossbred); ${p}_{\mathrm{ii}}$ is the purebred effect (PE) common to all progenies of mating between *i*th sire group and *j*th dam group; ${g}_{\mathrm{i}}$ (${g}_{\mathrm{j}}$) is the general combining ability (GCA) for the *i*th (*j*th) breed; ${m}_{\mathrm{j}}$ is the maternal effect (ME) of *j*th dam breed; ${s}_{\mathrm{ij}}$ is the specific combining ability (SCA) in progeny of *i*th and *j*th breed; ${r}_{\mathrm{ij}}$ is the residual reciprocal effect (RRE) in progeny of *i*th sire group and *j*th dam group; and ${e}_{\mathrm{hijk}}$ is the random error term.

## Results and discussion

### Relative performance

Tables 1 and 2 present the least-squares means (LSM) and the standard error (SE) of external and internal egg quality traits, respectively. Most internal (YW, AW, and YH) and all external egg quality traits were significantly (*P* < 0.001) different among the genotypes. Similarly, other authors (Al-Rawi and Amer 1972; Sola-Ojo and Ayorinde 2011; Khalil et al. 2013; Sinha et al. 2018; Hussen et al. 2019; Udoh et al. 2020; Wolde et al. 2021) found significant differences among different genotypes for most egg quality traits.

**Table 1:** Least-squares means (LSMs) for egg weight (EW), egg length (EL), egg width (EWD), shell weight (SW), shell thickness (ST), and egg shape index (ESI)

Trait	Age (Week)	Genotype (G)							SEM	*P* value		
		FM	KK	WL	FM x KK	FM x WL	KK x FM	WL x FM		G	Age (A)	G x A
EW, g	32	41.400^b^	46.958^a^	46.668^a^	46.399^a^	45.260^a^	45.735^a^	44.805^a^	3.265	< 0.0001	< 0.0001	0.8448
	36	42.085^c^	47.919^ab^	48.582^a^	47.122^ab^	46.120^ab^	47.372^ab^	44.758^bc^	3.670			
	40	43.639^c^	49.457^ab^	50.599^a^	47.904^ab^	46.911^b^	48.025^ab^	47.292^b^	3.836			
	Mean	42.375^d^	47.563^ab^	48.616^a^	47.142^abc^	46.097^bc^	47.044^abc^	45.618^c^	3.601			
EL, cm	32	4.793^b^	4.913^ab^	5.070^a^	5.047^a^	4.857^b^	4.960^ab^	4.970^ab^	0.244	< 0.0001	< 0.0001	0.5988
	36	4.913^c^	5.161^ab^	5.250^a^	5.110^ab^	5.093^ab^	5.017^bc^	5.137^ab^	0.228			
	40	5.093^c^	5.332^ab^	5.437^a^	5.300^ab^	5.213^bc^	5.190^bc^	5.273^abc^	0.254			
	Mean	4.933^c^	5.089^b^	5.252^a^	5.152^ab^	5.054^b^	5.056^b^	5.127^ab^	0.265			
EWD, cm	32	3.670^d^	3.997^a^	3.850^abc^	3.847^abc^	3.770^bcd^	3.863^ab^	3.740^ cd^	0.159	< 0.0001	< 0.0001	0.9978
	36	3.713^c^	3.950^a^	3.917^a^	3.850^ab^	3.820^abc^	3.897^a^	3.740^bc^	0.148			
	40	3.850^c^	4.071^a^	4.067^a^	3.997^ab^	3.977^abc^	4.063^a^	3.917^bc^	0.166			
	Mean	3.744^d^	3.956^a^	3.944^a^	3.898^ab^	3.856^bc^	3.941^a^	3.799^ cd^	0.182			
SW, g	32	4.272^abc^	3.817^c^	4.503^a^	4.101^bc^	4.531^a^	4.347^ab^	4.348^ab^	0.633	< 0.0001	< 0.0001	0.0563
	36	3.771^a^	3.881^a^	3.975^a^	4.044^a^	4.089^a^	3.710^a^	3.721^a^	0.610			
	40	4.691^ab^	4.687^ab^	4.928^a^	4.793^ab^	5.050^a^	4.475^b^	4.809^ab^	0.517			
	Mean	4.245^bc^	4.117^c^	4.469^ab^	4.313^abc^	4.557^a^	4.177^c^	4.293^abc^	0.626			
ST, mm	32	0.312^a^	0.268^c^	0.297^abc^	0.281^c^	0.308^ab^	0.286^bc^	0.295^abc^	0.031	< 0.0001	< 0.0001	0.0593
	36	0.255^ab^	0.214^c^	0.247^ab^	0.256^ab^	0.271^a^	0.238^bc^	0.238^bc^	0.034			
	40	0.340^ab^	0.278^d^	0.308^bcd^	0.307^bcd^	0.355^a^	0.299^ cd^	0.317^bc^	0.044			
	Mean	0.303^a^	0.264^c^	0.284^b^	0.282^b^	0.311^a^	0.274^b^	0.283^b^	0.039			
ESI, %	32	76.636^bc^	81.403^a^	76.061^bc^	76.263^bc^	77.682^bc^	77.943^ab^	75.343^c^	3.162	< 0.0001	0.0002	0.7679
	36	75.625^ab^	76.839^ab^	74.700^bc^	75.386^abc^	75.129^abc^	77.690^a^	72.968^c^	3.354			
	40	75.724^ab^	76.610^ab^	74.893^b^	75.452^ab^	76.392^ab^	78.600^a^	74.433^b^	4.189			
	Mean	75.995^bc^	78.058^ab^	75.218^ cd^	75.700^ cd^	76.401^bc^	78.078^a^	74.248^d^	3.647			

LSMs not connected by the same superscript in a row are significantly different at *P* < 0.05; In all the crosses, male and female parents are listed first and second, respectively; *FM* Fayoumi, *KK* Koekoek, *WL* White Leghorn, *SEM* Standard Error of the Mean

**Table 2:** Least-squares means (LSMs) for yolk weight (YW), albumen weight (AW), yolk height (YH), albumen height (AH), and Haugh unit (HU)

Trait	Age (Week)	Genotype (G)							SEM	*P* value		
		FM	KK	WL	FM x KK	FM x WL	KK x FM	WL x FM		G	Age (A)	G x A
YW, g	32	12.600^b^	14.088^ab^	13.867^ab^	14.358^a^	13.861^ab^	13.961^ab^	13.559^ab^	1.288	< 0.0001	< 0.0001	0.3436
	36	13.238^c^	14.342^abc^	14.831^ab^	15.309^a^	15.016^a^	15.028^a^	13.978^bc^	1.358			
	40	13.959^b^	15.424^a^	14.981^ab^	15.373^a^	14.518^ab^	15.217^a^	14.914^ab^	1.484			
	Mean	13.279^c^	14.555^ab^	14.571^ab^	15.024^a^	14.473^ab^	14.748^ab^	14.163^b^	1.383			
AW, g	32	23.455^b^	27.471^a^	27.389^a^	27.229^a^	25.979^a^	27.146^a^	25.740^a^	2.568	< 0.0001	< 0.0001	0.6716
	36	24.786^c^	28.832^ab^	29.540^a^	27.372^b^	27.003^b^	27.873^ab^	26.705^bc^	2.696			
	40	25.291^c^	29.132^ab^	30.738^a^	28.352^b^	27.631^b^	27.873^b^	27.544^b^	2.801			
	Mean	24.530^d^	28.770^ab^	29.255^a^	27.663^bc^	26.888^c^	27.640^bc^	26.682^c^	2.699			
YH, mm	32	14.287^b^	15.203^a^	14.630^ab^	14.860^ab^	14.660^ab^	14.917^a^	14.743^ab^	0.765	< 0.0001	< 0.0001	0.4408
	36	15.027^ab^	15.149^ab^	14.684^b^	15.464^a^	15.084^ab^	15.217^ab^	15.190^ab^	0.782			
	40	14.113^ab^	14.908^a^	13.927^b^	14.723^ab^	14.095^ab^	14.677^ab^	14.517^ab^	1.107			
	Mean	14.473^ cd^	15.088^a^	14.406^d^	15.014^a^	14.607^bcd^	14.933^ab^	14.814^abc^	0.899			
AH, mm	32	6.297^b^	6.406^ab^	6.760^ab^	6.310^b^	7.120^a^	6.567^ab^	6.460^ab^	1.007	0.0722	< 0.0001	0.1644
	36	7.340^a^	7.611^a^	7.333^a^	7.257^a^	7.267^a^	7.177^a^	7.167^a^	1.276			
	40	6.807^ab^	6.493^b^	7.577^a^	6.830^ab^	6.973^ab^	6.597^b^	7.113^ab^	1.202			
	Mean	6.819^a^	7.001^a^	7.230^a^	6.804^a^	7.118^a^	6.780^a^	6.919^a^	1.178			
HU	32	84.753^ab^	83.932^ab^	85.835^ab^	83.137^b^	88.285^a^	85.062^ab^	84.780^ab^	5.980	0.0561	< 0.0001	0.1683
	36	90.471^a^	90.179^a^	88.343^a^	88.318^a^	89.040^a^	87.665^a^	88.852^a^	7.016			
	40	87.046^ab^	82.877^b^	89.344^a^	85.669^ab^	86.845^ab^	84.252^ab^	87.612^ab^	6.953			
	Mean	87.444^a^	86.607^a^	87.868^a^	85.731^a^	88.041^a^	85.651^a^	87.105^a^	6.742			

LSMs not connected by the same superscript in a row are significantly different at *P* < 0.05; In all the crosses, male and female parents are listed first and second, respectively; *FM* Fayoumi, *KK* Koekoek, *WL* White Leghorn, *SEM* Standard Error of the Mean

In the current study, eggs of WL and KK were the heaviest, followed by FM x KK, KK x FM, FM x WL, WL x FM, and FM. Egg weight is an important trait that influences other egg quality traits. The WL, KK, KK x FM, and FM x KK had the highest EWD, followed by FM x KK, FM x WL, WL x FM, and FM. Eggs of WL, FM x KK, and WL x FM were the longest, with FM x WL, KK x FM, and KK having eggs of intermediate size and FM eggs being the shortest. There was the highest SW in FM x WL and WL, followed by FM x KK, WL x FM, FM, KK x FM, and KK. Eggs of FM x WL and FM had the thickest shell, while WL, WL x FM, FM x KK, and KK x FM showed medium ST, with eggs of KK having the thinnest shell. The KK x FM and KK showed the highest ESI, followed by FM, FM x WL, FM x KK, WL, and WL x FM. The higher the ESI is, the more uniform the eggs are, and uniformity of the eggs is essential for better hatchability and healthy chick production (Rajaravindra et al. 2015). According to the characterization of eggs based on shape index (Altuntaş and Şekeroğlu 2008; Ledvinka et al. 2012), most genotypes had eggs with normal (typical) shape, while KK x FM and KK had round eggs.

Of the internal quality traits, YW, AW, and YH showed significant (P < 0.001) differences among the genotypes (Table 2). The YW was higher in FM x KK, KK x FM, WL, KK, and FM x WL, with WL x FM and FM having the medium and the lowest YW, respectively. Eggs of WL and KK had the highest AW, followed by FM x KK, KK x FM, FM x WL, WL x FM, and FM. The present findings are in good agreement with the observation of Al-Rawi and Amer (1972), who reported that YW and AW were higher in heavier eggs. Eggs collected from KK and FM x KK showed the highest YH, followed by KK x FM, WL x FM, and FM x WL, with eggs of WL and FM having the lowest YH. Khawaja et al. (2013) reported that YW, AW, and AH were significantly superior in RIR than FM and its reciprocal crosses with RIR. The present results showed non-significant variation in AH among the genotypes, reflecting that eggs of all the genotypes have similar albumen quality.

Relative to FM, egg quality characteristics showed an improvement in most crossbreds. For instance, all the crossbreds outperformed FM in EW, EL, EWD, YW, and AW. In different studies (Khalil et al. 2013; Hussen et al. 2019; Udoh et al. 2020), crossbreds showed superior performances to their indigenous parents but not exotic ones for most traits. In most traits Al-Rawi and Amer (1972) studied, crossbreds performed better than exotic and indigenous parents. Sola-Ojo and Ayorinde (2011) also found higher EL, ST, AH, and AW in reciprocal crosses of Fulani (local chicken of Nigeria) and Dominant Black compared with exotic and local parents. Compared with both parents, HU, AH, and YH showed an improvement in reciprocal crosses of Horro (an improved Ethiopian local breed) and Dominant Red Barred (Hussen et al. 2019). These results generally suggest that crossbreeding could be used to enhance those traits. On the contrary, Sinha et al. (2018) observed significantly higher EW, EL, EWD, ST, SW, AH, AW, and HU in the two purebreds compared with the crossbreds; thus, the traits did not improve as a result of crossbreeding. The differences among the findings in exploiting heterosis would be related to the variations in gene frequency among the parental breeds involved.

Age affected all external and internal egg quality parameters (Table 1 and 2). Most quality traits were affected by the hens' age in various earlier studies (Rajaravindra et al. 2015; Samiullah et al. 2017; Wolde et al. 2021). Contrary to the current results, Wolde et al. (2021) reported significant genotype-by-age interaction effects on EW and EL. The absence of the interaction effects in the current study reflects that the same genotype did not show variation in egg quality traits across different ages.

### Additive genetic variance

#### Purebred effect

The PE was significantly (*P* < 0.001) different among the purebreds in all internal and external egg quality traits except SW (Table 3). For EW, EL, EWD, YW, and AW traits, the KK and WL had statistically similar, positive, and higher PE estimates, while FM showed negative and lower values. The higher PE was found in KK for EST and YH, followed by FM and WL, breeds that showed statistically similar and negatively lower estimates. The ST showed the highest, intermediate, and lowest PE values in FM (0.017), WL (− 0.002), and KK (− 0.027), respectively. According to Nath et al. (2007), the highest PE value suggests that the genotype could also have higher GCA or/and ME. As confirmed in the current study, most traits with higher PE in KK had higher GCA. The results also suggest the existence of favorable alleles and the importance of additive and dominant genetic variances for the inheritances of egg quality traits in the breed.

**Table 3:** Least-squares estimates and standard errors of different genetic effects for egg weight (EW), egg length (EL), egg width (EWD), shell weight (SW), shell thickness (ST), and egg shape index (ESI)

Genetic effect	EW	EL	EWD	SW	ST	ESI	YW	AW	YH
Overall mean, *μ*	46.298 ± 0.156	5.101 ± 0.011	3.874 ± 0.007	4.329 ± 0.024	0.287 ± 0.002	1.061 ± 0.001	14.337 ± 0.052	27.260 ± 0.119	14.709 ± 0.031
Heterosis,$\overline{{\varvec{h}} }$									
${{\varvec{a}}}_{1}$	− 0.177 ± 0.156	0.004 ± 0.011	0.001 ± 0.007	− 0.006 ± 0.024	− 0.001 ± 0.002	− 0.001 ± 0.001	− 0.254 ± 0.052	0.056 ± 0.119	− 0.136 ± 0.031
${{\varvec{a}}}_{2}$	0.177 ± 0.156	− 0.004 ± 0.011	− 0.001 ± 0.007	0.006 ± 0.024	0.001 ± 0.002	0.001 ± 0.001	0.254 ± 0.052	− 0.056 ± 0.119	0.136 ± 0.031
PE	***	***	***	NS	***	***	***	***	***
${{\varvec{p}}}_{11}$	− 3.746 ± 0.385^b^	− 0.172 ± 0.031^b^	− 0.131 ± 0.020^b^	− 0.079 ± 0.076^a^	0.017 ± 0.005^a^	− 0.001 ± 0.004^b^	− 0.818 ± 0.152^b^	− 2.804 ± 0.296^b^	− 0.097 ± 0.097^b^
${{\varvec{p}}}_{22}$	2.250 ± 0.517^a^	0.045 ± 0.042^a^	0.111 ± 0.027^a^	− 0.120 ± 0.101^a^	− 0.027 ± 0.007^c^	0.019 ± 0.006^a^	0.615 ± 0.205^a^	1.615 ± 0.398^a^	0.461 ± 0.131^a^
${{\varvec{p}}}_{33}$	2.496 ± 0.385^a^	0.147 ± 0.031^a^	0.069 ± 0.020^a^	0.145 ± 0.076^a^	− 0.002 ± 0.005^b^	− 0.010 ± 0.004^b^	0.476 ± 0.152^a^	1.907 ± 0.296^a^	− 0.159 ± 0.097^b^
GCA	**	NS	***	*	**	***	***	**	NS
${{\varvec{g}}}_{1}$	0.096 ± 0.374^ab^	0.004 ± 0.030^a^	0.002 ± 0.021^b^	0.067 ± 0.073^a^	0.006 ± 0.005^a^	− 0.001 ± 0.005^b^	0.099 ± 0.147^a^	0.038 ± 0.279^ab^	− 0.021 ± 0.097^a^
${{\varvec{g}}}_{2}$	1.203 ± 0.529^a^	− 0.019 ± 0.042^a^	0.107 ± 0.029^a^	− 0.225 ± 0.103^b^	− 0.022 ± 0.007^b^	0.029 ± 0.007^a^	0.474 ± 0.209^a^	0.867 ± 0.395^a^	0.235 ± 0.137^a^
${{\varvec{g}}}_{3}$	− 1.395 ± 0.529^b^	0.011 ± 0.042^a^	− 0.111 ± 0.029^c^	0.092 ± 0.103^a^	0.010 ± 0.007^a^	− 0.028 ± 0.007^c^	− 0.672 ± 0.209^b^	− 0.943 ± 0.395^b^	− 0.193 ± 0.137^a^
ME	NS	***	**	***	***	***	***	NS	NS
${{\varvec{m}}}_{1}$	− 0.192 ± 0.188^a^	− 0.008 ± 0.015^b^	− 0.004 ± 0.011^ab^	− 0.133 ± 0.036^b^	− 0.012 ± 0.002^c^	0.002 ± 0.002^b^	− 0.198 ± 0.074^b^	− 0.076 ± 0.140^a^	0.042 ± 0.048^a^
${{\varvec{m}}}_{2}$	0.065 ± 0.265^a^	0.064 ± 0.021^a^	− 0.029 ± 0.015^b^	0.090 ± 0.051^a^	0.005 ± 0.003^b^	− 0.020 ± 0.004^c^	0.185 ± 0.104^a^	0.013 ± 0.198^a^	0.053 ± 0.068^a^
${{\varvec{m}}}_{3}$	0.319 ± 0.265^a^	− 0.048 ± 0.021^b^	0.038 ± 0.015^a^	0.176 ± 0.051^a^	0.019 ± 0.003^a^	0.017 ± 0.004^a^	0.210 ± 0.104^a^	0.139 ± 0.198^a^	− 0.136 ± 0.068^a^
SCA	***	NS	***	**	***	***	***	***	***
${{\varvec{s}}}_{12}$	− 0.618 ± 0.192^b^	− 0.007 ± 0.014^a^	− 0.046 ± 0.010^b^	0.090 ± 0.038^a^	0.010 ± 0.003^a^	− 0.010 ± 0.002^b^	− 0.283 ± 0.070^b^	− 0.437 ± 0.152^b^	− 0.131 ± 0.052^b^
${{\varvec{s}}}_{13}$	0.618 ± 0.192^a^	0.007 ± 0.014^a^	0.046 ± 0.010^a^	− 0.090 ± 0.038^b^	− 0.010 ± 0.003^b^	0.010 ± 0.002^a^	0.283 ± 0.070^a^	0.437 ± 0.152^a^	0.131 ± 0.052^a^
RRE	NS	NS	NS	NS	NS	**	NS	NS	NS
${{\varvec{r}}}_{12}$	− 0.080 ± 0.192^a^	0.012 ± 0.014^a^	− 0.009 ± 0.010^a^	− 0.044 ± 0.038^a^	− 0.005 ± 0.003^a^	− 0.004 ± 0.002^b^	− 0.053 ± 0.070^a^	− 0.035 ± 0.152^a^	0.034 ± 0.052^a^
${{\varvec{r}}}_{13}$	− 0.016 ± 0.192^a^	− 0.016 ± 0.014^a^	0.007 ± 0.010^a^	− 0.023 ± 0.038^a^	− 0.001 ± 0.003^a^	0.005 ± 0.002^a^	− 0.046 ± 0.070^a^	− 0.003 ± 0.152^a^	− 0.013 ± 0.052^a^

Estimates not connected by the same superscript in a column are significantly different at *P* < 0.05; *NS* not significant, * P < 0.05, ** P < 0.01, *** P < 0.001; the subscripts 1, 2, and 3 indicate FM, KK, and WL parental breeds, respectively; the subscripts 12 and 13 represent FM x KK and FM x WL crossbreds, respectively

#### General combining ability

The GCA, the numeric value that indicates the influence of one of the breeds on its crossbred progeny, showed significant variation among the breeds in all external egg quality traits except EL and two internal quality traits (YW and AW; Table 3), which suggests the importance of additive effects for the inheritance of those traits. For YW, AW, and EW, the KK, and FM exhibited the highest and positive values, while WL had the lowest and negative GCA. The KK had the highest GCA in ESI, followed by FM and WL, which showed negative values. However, the reverse is true for SW and ST, where FM and WL showed statically similar and higher positive values, while KK had a lower and negative GCA. The positive and highest GCA value in KK for most traits indicates the accumulation of favorable alleles in the breed. The desirable genes accumulated in KK would likely be passed on to the crosses having this breed as a parent (i.e., good combining ability).

#### Maternal effect

Significant variation in ME was found in all external egg quality traits except EW and only in one internal quality trait (YW; Table 3), which suggests the importance of maternal additive and dominance gene effects for the variation among the breeds in egg quality characteristics. For YW and SW, WL and KK breeds had statistically similar and higher values, while FM showed a lower and negative estimate. The KK had positive and higher ME than FM (− 0.008) and WL (− 0.048) for EL. The highest, intermediate, and lowest values were found in WL, FM, and KK, respectively, for EWD and ESI traits. Both WL (0.176) and KK (0.090) had statistically similar and higher ME for SW, while FM showed a lower estimate (− 0.133). The ST trait showed higher, medium, and lower values in WL (0.019), KK (0.005), and KK (− 0.012). It would be suitable to use breeds with higher ME as a mother line to develop hybrids having higher egg quality characteristics.

### Non-additive genetic variance

#### Specific combining ability

Variation due to SCA, the numeric value that measures the average inferiority or superiority of particular crosses relative to the average performance of their parental breeds (Henderson 1948; Harvey 1960; Falconer and Mackay 1996), was significant in all internal (*P* < 0.001) and all external egg quality traits except EL (Table 3). The results suggest the importance of non-additive effects for the inheritance of egg quality parameters. For EW, EWD, ESI, YW, AW, and YH traits, FM x WL exhibited positive and higher values, but FM x KK had negative and lower SCA, which suggests that crossing FM with WL would be more beneficial than crossing it with KK to exploit non-additive gene effects. The reverse is true for SW and ST, where FM x KK showed positive and higher values, while the values were negative and lower in FM x WL.

#### Residual reciprocal effect

The RRE variation was significant (*P* < 0.001) only in ESI, where FM x WL had a higher and positive value, but FM x KK showed a lower and negative estimate. The results suggest that genetic interaction with sex chromosomes is not important for the inheritance of egg quality traits.

In the present study, all egg quality traits except AH and HU differed significantly among the genotypes. Most egg quality traits showed superior performance in crossbreds relative to FM, and some crossbreds outperformed both parents. The results also revealed significant variation due to PE, GCA, ME, and SCA, which suggest that additive and non-additive genetic variances are crucial for egg quality traits to be inherited. Therefore, genetic improvement in the genotypes investigated would be possible using selection and crossbreeding strategies and/or a combination of the two.

## Data Availability

The data that support the findings of this study are included in the manuscript but are available from the corresponding author on reasonable request.

## References

[CR1] Adeleke MA, Peters SO, Ozoje MO, Ikeobi CON, Bamgbose AM, Adebambo OA (2012). Effect of crossbreeding on fertility, hatchability and embryonic mortality of Nigerian local chickens. Tropical Animal Health and Production.

[CR2] Adem SA (2021). Impacts of rainfall variability on potato productivity in Haramaya district, eastern Hararge zone, Ethiopia. Journal of Chemical, Environmental and Biological Engineering.

[CR3] Al-Rawi BA, Amer MF (1972). Egg quality of some purebred chickens and their crosses in the subtropics. Poultry Science.

[CR4] Altuntaş E, Şekeroğlu A (2008). Effect of egg shape index on mechanical properties of chicken eggs. Journal of Food Engineering.

[CR5] Bekele F, Ådnøy T, Gjøen HM, Kathle J, Abebe G (2010). Production performance of dual purpose crosses of two indigenous with two exotic chicken breeds in sub-tropical environment. International Journal of Poultry Science.

[CR6] Besbes B (2009). Genotype evaluation and breeding of poultry for performance under suboptimal village conditions. World's Poultry Science Journal.

[CR7] Box GPE, Cox DR (1964). An analysis of transformations. Journal of the Royal Statistical Society: Series B (methodological).

[CR8] Burga S, Tesfaye K, Dechassa N, Tana T, Mohammed W (2020). Trends in observed temperature and rainfall variability in major potato growing districts of eastern Ethiopia. Ethiopian Journal of Crop Science.

[CR9] CSA, 2021. Agricultural Sample Survey 2020/21 (2013 E.C.): Report on Livestock and Livestock Characteristics. Statistical Bulletin No. 589, Vol. II, Addis Ababa. http://www.statsethiopia.gov.et/wp-content/uploads/2021/05/2013.LIVESTOCK-REPORT.FINAL_.pdf. Accessed 20 Jan 2022.

[CR10] Falconer DS, Mackay TFC (1996). Introduction to Quantitative Genetics.

[CR11] FAO, 2019. Poultry Sector Ethiopia. FAO animal production and health livestock country reviews No. 11, Rome. https://www.fao.org/3/ca3716en/ca3716en.pdf. Accessed 4 Nov 2022.

[CR12] Galukande, E., Mulindwa, H., Wurzinger, M., Roschinsky, R., Mwai, A.O. and Sölkne, J., 2013. Crossbreeding cattle for milk production in the tropics: Achievements, challenges and opportunities. Animal Genetic Resources, 52, 111–125.

[CR13] Geleta T, Abdulkadir U (2018). Production performance evaluation of Koekoek chickens at Adami Tulu research centre. African Journal of Agricultural Research.

[CR14] Geleta T, Leta S, Bekana E (2013). Production performance of Fayoumi chickens under intensive management condition of Adami Tulu research centre. International Journal of Livestock Production.

[CR15] Harvey WR (1960). Least-squares Analysis of Data with Unequal Subclass Numbers.

[CR16] Haugh RR (1937). The Haugh unit for measuring egg quality. United States Egg and Poultry Magazine.

[CR17] Henderson, C. R., 1948. Estimation of general, specific, and maternal combing abilities in crosses among inbred lines of swine, (unpublished PhD thesis, Iowa State College). https://lib.dr.iastate.edu/rtd/12780. Accessed 15 Sept 2020.

[CR18] Hussen K, Goshu G, Esatu W, Abegaz S (2019). Comparing egg quality traits of crossbred local Horro ecotype with Dominant Red Bared D 922 exotic chickens: a step towards synthetic breed development in Ethiopia. British Journal of Poultry Sciences.

[CR19] Jakubec V, Komender P, Nitter G, Fewson D, Soukupova Z (1987). Crossbreeding in farm animals. I. Analysis of complete diallel experiments by means of three models with application to poultry. Journal of Animal Breeding and Genetics.

[CR20] Khalil, M.H., Iraqi, M.M. and El-Atrouny, M.M., 2013. Effects on egg quality traits of crossing Egyptian Golden Montazah with White Leghorn chickens. Livestock Research for Rural Development, 25(6).

[CR21] Khawaja T, Khan SH, Mukhtar N, Ullah N, Parveen A (2013). Production performance, egg quality and biochemical parameters of Fayoumi, Rhode Island Red and their reciprocal crossbred chickens. Journal of Applied Animal Research.

[CR22] Ledvinka Z, Zita L, Klesalová L (2012). Egg quality and some factors influencing it: a review. Scientia Agriculturae Bohemica.

[CR23] Lemlem, A. and Tesfay, Y., 2010. Performance of exotic and indigenous poultry breeds managed by smallholder farmers in northern Ethiopia. Livestock Research for Rural Development, 22(7).

[CR24] Leroy, G., Baumung, R., Boettcher, P., Scherf, B. and Hoffmann, I., 2016. Review: Sustainability of crossbreeding in developing countries; definitely not like crossing a meadow…. Animal, 10(2), 262–273. 10.1017/S175173111500213X 26503101

[CR25] Munisi, W.G., Katule, A.M. and Mbaga, S.H., 2015. Comparative growth and livability performance of exotic, indigenouschickens and their crosses in Tanzania. Livestock Research for Rural Development, 27(4).

[CR26] Nath M, Singh BP, Saxena VK, Singh RV (2007). Analyses of crossbreeding parameters for juvenile body weight in broiler chicken. Journal of Applied Animal Research.

[CR27] Negash, F., Abegaz, S., Tadesse, Y., Jembere, T., Esatu, W. and Dessie, T., 2023. Evaluation of growth performance and feed efficiency in reciprocal crosses of Fayoumi with three exotic chicken breeds. Acta Agriculturae Scandinavica, Section A — Animal Science. 10.1080/09064702.2023.2168743.

[CR28] NRC, 1994. Nutrient Requirements of Poultry. National Research Council. 9th revised ed., (National Academies Press, USA). https://nap.nationalacademies.org/catalog/2114/nutrient-requirements-of-poultry-ninth-revised-edition-1994. Accessed Jan 2020.

[CR29] Rajaravindra KS, Rajkumar U, Rekha K, Niranjan M, Reddy BLN, Chatterjee RN (2015). Evaluation of egg quality traits in a synthetic coloured broiler female line. Journal of Applied Animal Research.

[CR30] Samiullah S, Omar AS, Roberts J, Chousalkar K (2017). Effect of production system and flock age on eggshell and egg internal quality measurements. Poultry Science.

[CR31] SAS Institute Inc., 2018. JMP® Version 14. SAS Institute Inc., Cary, NC: SAS Institute Inc. https://www.jmp.com.

[CR32] Senbeta EK (2017). Growth performance and rearing costs of Fayoumi and White Leghorn chicken breeds. East African Journal of Sciences.

[CR33] Senbeta EK, Balcha KA (2020). Performance of White Leghorn chickens breed maintained at Haramaya University poultry farm and implications for sustainable poultry production. East African Journal of Sciences.

[CR34] Simm G, Pollott G, Mrode R, Houston R, Marshall K (2021). Genetic Improvement of Farmed Animals.

[CR35] Sinha B, Mandal KG, Kumari R, Kumari A, Gonge DS (2018). Genetic impact on external and internal egg quality traits of Vanaraja and Gramapriya birds and their crosses in Bihar. Indian Journal of Animal Research.

[CR36] Sola-Ojo FE, Ayorinde KL (2011). Evaluation of reproductive performance and egg quality traits in progenies of Dominant Black strain crossed with Fulani ecotype chicken. Journal of Agricultural Science.

[CR37] Tadelle D, Alemu Y, Peters KJ (2000). Indigenous chickens in Ethiopia: genetic potential and attempts at improvement. World's Poultry Science Journal.

[CR38] Tadesse D, Singh H, Mengistu A, Esatu W, Dessie T (2013). Study on productive performances and egg quality traits of exotic chickens under village production system in East Shewa. Ethiopia. African Journal of Agricultural Research.

[CR39] Udoh JE, Udoh UH, Adeoye AA (2020). Comparative analysis of fertile egg quality traits in exotic, two Nigerian ecotype chickens and their crosses. Nigerian Journal of Animal Science.

[CR40] Williams SM, Price SE, Siegel PB (2002). Heterosis of growth and reproductive traits in fowl. Poultry Science.

[CR41] Wolde S, Mirkena T, Melesse A, Dessie T, Abegaz S (2021). Egg production and quality traits of Sasso-RIR, normal feathered local and their F1 cross chickens managed under on-station condition in southern Ethiopia. Tropical and Subtropical Agroecosystems.

[CR42] Wolf, J. and Knížetová, H., 1994. Crossbreeding effects for body weight and carcass traits in Pekin duck. British Poultry Science, 35(1), 33–45. 10.1080/00071669408417668 8199889

[CR43] Wolf, J., Nitter, G., Fewson, D., Jakubec, V. and Soukupová, Z., 1991. Crossbreeding in farm animals. IV. Analysis of partial diallels with two sets of parental populations. Journal of Animal Breeding Genetics, 108, 23–34.

[CR44] Wondmeneh E, van Der Waaij EH, Tadelle D, Udo HMJ, van Arendonk JAM (2014). Adoption of exotic chicken breeds by rural poultry keepers in Ethiopia. Acta Agric Scand, Sect A — Anim Sci.

